# Relative contributions of environmental pollen exposure and host susceptibility to *Artemisia*-related sensitisation and allergy in a high-exposure region of Northern China: a population-based cross-sectional study

**DOI:** 10.3389/fimmu.2026.1887300

**Published:** 2026-07-23

**Authors:** Le Cui, Lun Li, Junda Li, Shuang Liu, Xin Li, Ruoyu Ji, Wendong Hao, Bin Wu, Huanhuan Wang, Yanhong Wang, Zhenxing Zheng, Jia Yin

**Affiliations:** 1 Department of Allergy, Peking Union Medical College Hospital, Chinese Academy of Medical Sciences & Peking Union Medical College, Beijing, China; 2 Beijing Key Laboratory of Precision Medicine for Diagnosis and Treatment on Allergic Diseases, Beijing, China; 3 National Clinical Research Center for Dermatologic and Immunologic Diseases, Beijing, China; 4 The First Affiliated Hospital of Xi’an Jiaotong University, Yulin Hospital, Yulin, China; 5 Yulin City Center for Disease Control and Prevention (Yulin Health Supervision Institute), Yulin, China; 6 Institute of Basic Medical Sciences & School of Basic Medicine, Chinese Academy of Medical Sciences & Peking Union Medical College, Beijing, China

**Keywords:** acute asthma exacerbation, allergic rhinitis, *Artemisia* pollen, sensitization, allergy, prevalence, influencing factors

## Abstract

**Objective:**

*Artemisia* pollen is a major airborne allergen in northern China; however, the relative contributions of environmental pollen exposure and host susceptibility to allergic disease remain unclear in high-exposure regions.

**Methods:**

A population-based cross-sectional study was conducted in Yulin City, China, involving 12,345 participants recruited through multistage stratified cluster sampling. Serum *Artemisia*-specific IgE was measured in 3,130 participants. Disease status was classified into healthy, sensitized, allergic rhinitis, and acute asthma exacerbation groups. Regional pollen exposure was categorized into low, medium, and high levels based on monitoring data. Inverse probability weighting was applied to reduce selection bias. Multivariable logistic regression models were used to assess associations.

**Results:**

The IPW-adjusted prevalence of *Artemisia* sensitization, allergic rhinitis, and acute asthma exacerbations was 13.0%, 10.7%, and 1.9%, respectively. Regional pollen concentration was not significantly associated with sensitization or disease outcomes in adjusted models (all P > 0.05). In contrast, host-related factors, including family history of allergy and *Artemisia*-specific IgE levels, showed consistent associations across disease stages. Family history of allergy was strongly associated with sensitization (OR = 4.57, 95% CI: 3.01–6.95). Increasing *Artemisia*-specific IgE levels were associated with higher odds of progression from sensitization to clinical disease and from rhinitis to acute asthma exacerbation.

**Conclusion:**

In this high-exposure population, host susceptibility factors demonstrated stronger and more consistent statistical associations with *Artemisia*-related allergic disease than regional pollen concentration. These findings suggest that host heterogeneity may contribute importantly to disease variability in high-exposure environments; however, causal inference is limited by the cross-sectional design and exposure measurement constraints.

## 1 Introduction

Allergic rhinitis (AR) is an immunoglobulin E (IgE)-mediated inflammatory disease of the nasal mucosa, characterized by nasal itching, sneezing, rhinorrhea, and nasal congestion. The disease is recurrent and persistent, significantly impairing patients’ quality of life and markedly increasing the risk of asthma development. In recent years, the prevalence of pollen allergy has been rising globally, particularly in highly industrialized countries. A multinational European study indicated that seasonal (intermittent) allergic rhinitis is the most common clinical phenotype in practice, with its prevalence substantially higher than that of the persistent type (1, 2). Additionally, a large, nationally representative survey in the United States (NHANES) reported that the sensitization rate to major weed pollen allergens, such as ragweed, was approximately 25.9% (3), reflecting the widespread nature of pollen sensitization in that country. Data from Japan indicate that by 2019, the overall prevalence of pollinosis had reached 38.8%, with the proportion of Japanese cedar pollinosis increasing steadily (4). In China, pollen allergy affects approximately 30% of patients with allergic rhinitis, involving over 75 million individuals, and has become a major public health problem affecting residents in northern China (5).

In northern China, *Artemisia* pollen is one of the most important sensitizing pollens, widely distributed in arid and semi-arid climatic zones (6). Its pollen grains are small, can be dispersed over long distances, and are highly allergenic. During summer and autumn, airborne *Artemisia* pollen concentrations increase significantly, making it a major trigger for seasonal allergic rhinitis (SAR) and an important contributor to asthma symptoms (7, 8).

Yulin City is located in northern Shaanxi Province, at the transition zone between the Mu Us Desert and the Loess Plateau. It has a typical temperate semi-arid climate that is dry and windy, with vegetation dominated by *Artemisia* species (6, 9). According to local pollen monitoring data (10), *Artemisia* pollen accounts for the majority of the total annual pollen count in Yulin during the summer–autumn period, confirming its dominance among local aeroallergens. Monitoring data show a distinct bimodal distribution of local pollen concentrations: July to October is the peak period for herbaceous pollen (predominantly *Artemisia*), while March to May is a secondary peak for tree pollen; pollen concentrations are lower during other periods (10). This ecological profile establishes Yulin as a typical high-incidence area for autumn and summer pollinosis in northern China, where pollen-related AR and asthma exhibit marked seasonality.

Although pollen allergy in Yulin has attracted growing attention, systematic research on *Artemisia* pollen remains insufficient. There is currently a lack of systematic epidemiological evidence regarding the risks of sensitization, allergy, and acute asthma exacerbation associated with *Artemisia* pollen. Our previous research indicated a significant dose-dependent relationship between *Artemisia*-specific IgE level and asthma risk, with higher specific IgE levels (e.g., ≥50 kU/L) associated with a substantially increased risk of asthma development (11). However, the key factors associated with the sequence from sensitization to allergic rhinitis and further to asthma remain unclear, particularly the epidemiological characteristics in high-exposure regions of China, which urgently need clarification.

This study conducted a large-scale population survey in Yulin City, a high-exposure area for *Artemisia* pollen, using questionnaires and serological testing to assess the sensitization rate to *Artemisia* pollen, the prevalence of AR, and the characteristics of acute asthma exacerbations, and to explore the relative importance of pollen exposure versus host factors in disease status. The findings will provide a basis for the prevention and control of pollen allergic diseases in northern China and serve as a reference for epidemiological studies in similar ecological regions.

## 2 Methods

### 2.1 Study design

This study was a population-based cross-sectional epidemiological survey led by the Department of Allergy at Peking Union Medical College Hospital, in collaboration with the Yulin Municipal Health Commission, Yulin Municipal Center for Disease Control and Prevention, and Yulin Second Hospital. The survey was conducted simultaneously across all 12 counties (districts) of Yulin City from June to September 2023, during the peak pollen season. The study aimed to systematically evaluate the prevalence and characteristics of *Artemisia* pollen-induced AR and acute asthma exacerbation, and to explore the relative importance of environmental versus host factors in the process from sensitization to AR and acute asthma exacerbation in the general population of Yulin.

### 2.2 Study population and sampling

A stratified multistage cluster random sampling strategy was employed. Stratification was first performed based on the latest urban-rural population structure of Yulin City (urban population: 61.6%, rural population: 38.4%). In the first stage, 14 investigation units (9 urban streets and 5 rural townships) were randomly selected. In the second stage, one community (urban) or administrative village (rural) was randomly chosen from each selected unit as the final survey site. All included participants were permanent residents who had lived continuously in the sampled area for at least six months.

### 2.3 Sample size estimation

The sample size calculation was based on data from the China Pulmonary Health (CPH) study (12), which reported an asthma prevalence of 4.2% among Chinese adults aged 20 years and above, with 15.5% of asthmatic patients having at least one emergency department visit due to acute asthma exacerbation in the past year. From this, the expected prevalence (P) of emergency visits for acute asthma exacerbations in the general population was estimated to be 0.65% (P = 0.0065). With a two-sided α of 0.05, a margin of error (d) of 0.002, and a design effect (deff) of 2.0 to account for the complex sampling design, the sample size was calculated using the formula for cross-sectional studies: n = [(Z² × P × (1−P))/d²] × deff = [(1.96² × 0.0065 × 0.9935)/0.002²] × 2.0 ≈ 12,400. The calculation indicated a required sample size of 12,400 individuals. Based on the total sample size, each of the 14 survey sites needed to recruit approximately 886 participants on average. This study initially enrolled 12,423 respondents. After excluding participants with incomplete questionnaire data or missing key variables (n=78), 12,345 respondents were included in the final analysis.

### 2.4 Variable definitions and classification

According to the July–October 2023 pollen data from the Yulin Meteorological Bureau, pollen sampling was performed using the gravity sedimentation method according to the China Meteorological Administration standard QX/T 324-2016. Areas were categorized into three groups based on mean pollen concentration: low (≤100 grains/thousand mm²), medium (101–150 grains/thousand mm²), and high (≥151 grains/thousand mm²).

Parental education level was defined as the higher educational attainment of either parent, categorized as low (primary school or below), medium (junior or senior high school), and high (college or above). Based on symptom frequency and functional impact as described in the ARIA guidelines, rhinitis severity was reclassified into three modified grades: mild (slight disturbance in one aspect of daily life), moderate (noticeable impairment of two to three daily functions), and severe (marked interference with sleep, daily activities and work/study). Family history of allergy was defined as self-reported physician-diagnosed allergic disease (asthma, allergic rhinitis, or atopic dermatitis) in at least one first-degree relative.

Rhinitis: Defined as self-report of at least two of the following nasal symptoms (nasal itching, sneezing, rhinorrhea, nasal congestion) occurring within the past 12 months, with each symptom episode lasting for more than one hour.

Acute asthma exacerbation: Defined as self-report of an emergency department visit or hospitalization due to sudden worsening of asthma symptoms (wheezing, chest tightness, dyspnea, or coughing) within the past 12 months.

Symptomatic: Defined as having either rhinitis or acute asthma exacerbation as defined above.

Seasonal symptomatic: Defined as report of rhinitis symptoms or acute asthma exacerbation occurring or with marked worsening during the summer–autumn period (predominantly from July to October coinciding with the *Artemisia* pollen peak season).

Based on the two dimensions of “symptom status” and “*Artemisia* sIgE status”, the study population was classified into the following categories: (1) Healthy: individuals who were asymptomatic and had negative *Artemisia* sIgE (< 0.35 kUA/L); (2) Sensitization: individuals who were asymptomatic but had positive *Artemisia* sIgE (≥ 0.35 kUA/L), reflecting “biological sensitization” without clinical expression; (3) Allergy (*Artemisia*-induced acute asthma exacerbation or allergic rhinitis): individuals who were seasonal symptomatic and had positive *Artemisia* sIgE (≥ 0.35 kUA/L), representing allergy with clinical disease manifestation. Symptomatic individuals with negative *Artemisia* sIgE were excluded from this study, as their symptoms might be attributable to other allergens or non-allergic factors.

Based on the above classifications, three multivariate logistic regression models were constructed: (i) Sensitization vs. Healthy; (ii) Allergy vs. Sensitization; and (iii) *Artemisia*-induced acute asthma exacerbation vs. allergic rhinitis.

### 2.5 Serological testing and sensitization assessment

This study planned to collect 3,000 serum samples, aiming to include approximately 2,000 respondents with symptoms of rhinitis or a history of acute asthma exacerbation, and 1,000 asymptomatic controls. Based on the total sample size requirement, the planned collection was allocated across the 14 survey sites, with each site aiming for approximately 214 serum samples. The electronic questionnaire system automatically triggered a blood sampling prompt when respondents reported rhinitis symptoms, a history of acute asthma exacerbation, or the absence of relevant symptoms. Ultimately, valid serum samples were successfully collected and analyzed from 3,135 respondents.

Specific IgE was measured using the ‌AllergyScreen immunoblot assay (‌Mediwiss Analytic GmbH) for 30 common aeroallergens. A specific IgE level ≥0.35 kUA/L was considered positive for sensitization and was further quantified and graded into classes 1–6 according to the following intervals: Class 1: ≥0.35–0.70 kUA/L; Class 2: ≥0.70–3.50 kUA/L; Class 3: ≥3.50–17.50 kUA/L; Class 4: ≥17.50–50.00 kUA/L; Class 5: ≥50.00–100.00 kUA/L; and Class 6: ≥100.00 kUA/L..

### 2.6 Data collection and quality control

A standardized electronic questionnaire system designed by Peking Union Medical College Hospital was used for data collection, with trained investigators providing on-site guidance to respondents. All respondents completed the questionnaire online via electronic devices. The system incorporated built-in logic checks and real-time review functions to ensure data completeness and accuracy. To ensure study quality, a subcenter implementation system was established, with district/county Centers for Disease Control and Prevention serving as sub-centers responsible for field investigation. The expert committee of Peking Union Medical College Hospital and Yulin City Center for Disease Control and Prevention jointly developed unified standard operating procedures (SOPs) and conducted centralized training for key personnel from each sub-center. Subsequently, the sub-centers conducted secondary training for the field investigators. Each survey site was equipped with epidemiology professionals and clinical physicians responsible for on-site review of data logical consistency and completeness.

### 2.7 Statistical analysis

All statistical analyses were performed using SPSS software. In descriptive analyses, quantitative data were presented as mean ± standard deviation, and categorical data as frequency (percentage). Prevalence rates of sensitization, AR, and acute asthma exacerbations were calculated along with their 95% confidence intervals (CIs), and comparisons were made between urban/rural areas and pollen concentration strata. In inferential analyses, continuous variables were analyzed using t-tests or ANOVA, and categorical variables using the χ² test or Fisher’s exact test. Multivariate logistic regression was used to analyze risk factors for AR and acute asthma exacerbations, calculating adjusted odds ratios (ORs) and 95% CIs. Log-transformed continuous *Artemisia*-specific IgE values (Log *Artemisia*-sIgE) were used in the logistic regression models to avoid the assumption of equal intervals between grades. Values below the detection limit (<0.35 kUA/L) were replaced with half the detection limit (0.175 kUA/L) before transformation. To correct for potential selection bias introduced by blood sampling, inverse probability weighting (IPW) was employed. A logistic regression model was first built based on the baseline characteristics of the participants who provided blood samples (including age, sex, urban/rural distribution, pollen concentration stratum, family history, symptomatic allergy) to predict the probability of blood sampling and calculate individual inverse probability weights. Subsequently, prevalence rates of sensitization, AR, and acute asthma exacerbations were estimated using the weighted dataset to reduce the impact of sampling bias on the results. The standardized mean difference (SMD) was used to assess covariate balance before and after IPW. After weighting (**Supplementary Table 1**), all covariates achieved good balance (|SMD| < 0.1), indicating effective correction of selection bias. Exploratory piecewise linear regression analyses were also performed using a pollen concentration cut point of 150 grains/thousand mm² to assess potential threshold effects, with results presented in **Supplementary Table 2**.

### 2.8 Ethics statement

The study protocol was approved by the Ethics Review Committee of Peking Union Medical College Hospital (approval number: I-23PJ536). All adult participants provided written informed consent before participation. For minors, written informed consent was provided by their legal guardians or parents.

## 3 Results

A total of 12,423 respondents were recruited in this study. After excluding 78 incomplete surveys, 12,345 individuals were included in the final statistical analysis. The study population consisted of 5,711 males (46.3%) and 6,634 females (53.7%), with a mean age of 38.33 years (range: 3–79 years). Among them, 7,536 (61.0%) were urban residents and 4,809 (39.0%) were rural residents. According to pollen exposure levels, 3,424 individuals (27.7%) resided in low pollen concentration areas, 3,769 (30.5%) in medium pollen concentration areas, and 5,152 (41.7%) in high pollen concentration areas.

### 3.1 Prevalence and distribution characteristics of rhinitis and acute asthma exacerbations

Among the 12,345 respondents, 8,949 (72.5%) reported no symptoms, 3,387 (27.4%) were classified as having rhinitis, and 400 (3.2%) had acute asthma exacerbations, of whom 391 (97.8%) also presented with rhinitis symptoms. Analysis of rhinitis severity showed that the proportion of patients with moderate-to-severe rhinitis in the acute asthma exacerbation group (81.8%) was significantly higher than that in the rhinitis-only group (53.5%), suggesting a close association between rhinitis severity and the risk of acute asthma exacerbations.

Patients with acute asthma exacerbations had an average of 2.17 emergency visits and 2.93 hospitalizations per year due to asthma or dyspnea. Overall, 43.8% of all patients (175/400) required oral or injected corticosteroids for asthma management, while among the subgroup sensitized to *Artemisia* pollen, this proportion was higher, reaching 49% (73/149). The blood sampling rates across groups were as follows: acute asthma exacerbation group, 73.8% (295/400); rhinitis group, 61.3% (2,076/3,387); and asymptomatic group, 11.7% (1,048/8,949).

**Table 1** shows the distribution differences among the three groups in terms of demographic characteristics, environmental exposure, and other allergy risk factors. The proportions of urban residents in the rhinitis and acute asthma exacerbation groups (69.7% and 78.3%, respectively) were significantly higher than that in the asymptomatic group (57.8%). Regarding pollen exposure area distribution, the proportions of residents in high pollen concentration areas were similar across the three groups: 42.0% in the asymptomatic group, 41.0% in the rhinitis group, and 40.5% in the acute asthma exacerbation group. Regarding age composition, the asymptomatic group had the highest mean age (39.48 ± 20.37 years), while the rhinitis and acute asthma exacerbation groups were relatively younger (35.30 ± 17.59 years and 34.41 ± 17.63 years, respectively). The positive rates of family history of allergy in the rhinitis and acute asthma exacerbation groups (35.2% and 45.0%, respectively) were significantly higher than that in the asymptomatic group (8.1%). Parental education level also showed a clear gradient: low education decreased from 59.6% in asymptomatic subjects to 45.4% in rhinitis and 39.3% in acute asthma exacerbations, while medium education increased from 24.0% to 31.1% to 34.5%, and high education from 16.4% to 23.4% to 26.3%.

**Table 1:** Distribution characteristics of asymptomatic, rhinitis, and acute asthma exacerbation groups.

Characteristic	Subgroup	Asymptomatic (N = 8,949)	Rhinitis (N = 3,387)	Acute asthma exacerbation (N = 400)	Total (N = 12,345)
Pollen concentration	low	2407 (26.9%)	1014 (29.9%)	147 (36.8%)	3424 (27.7%)
	medium	2781 (31.1%)	986 (29.1%)	91 (22.8%)	3769 (30.5%)
	High	3761 (42.0%)	1387 (41.0%)	162 (40.5%)	5152 (41.7%)
Residence	Rural	3,779 (42.2%)	1,026 (30.3%)	87 (21.8%)	4,809 (39.0%)
	Urban	5,170 (57.8%)	2,361 (69.7%)	313 (78.3%)	7,536 (61.0%)
Age (years)	Mean (SD)	39.48 (20.37)	35.30 (17.59)	34.41 (17.63)	38.33 (19.74)
Sex	Male	4,227 (47.2%)	1,478 (43.6%)	164 (41.0%)	5,711 (46.3%)
	Female	4,722 (52.8%)	1,909 (56.4%)	236 (59.0%)	6,634 (53.7%)
Parental education	Low	5,330 (59.6%)	1,538 (45.4%)	157 (39.3%)	6,873 (55.7%)
	Medium	2,148 (24.0%)	1,055 (31.1%)	138 (34.5%)	3,205 (26.0%)
	High	1,471 (16.4%)	794 (23.4%)	105 (26.3%)	2,267 (18.4%)
Family history of allergy	No	8,226 (91.9%)	2,194 (64.8%)	220 (55.0%)	10,428 (84.5%)
	Yes	723 (8.1%)	1,193 (35.2%)	180 (45.0%)	1,917 (15.5%)

### 3.2 Allergen sensitization profile and seasonal distribution of symptoms

**Figure 1:**
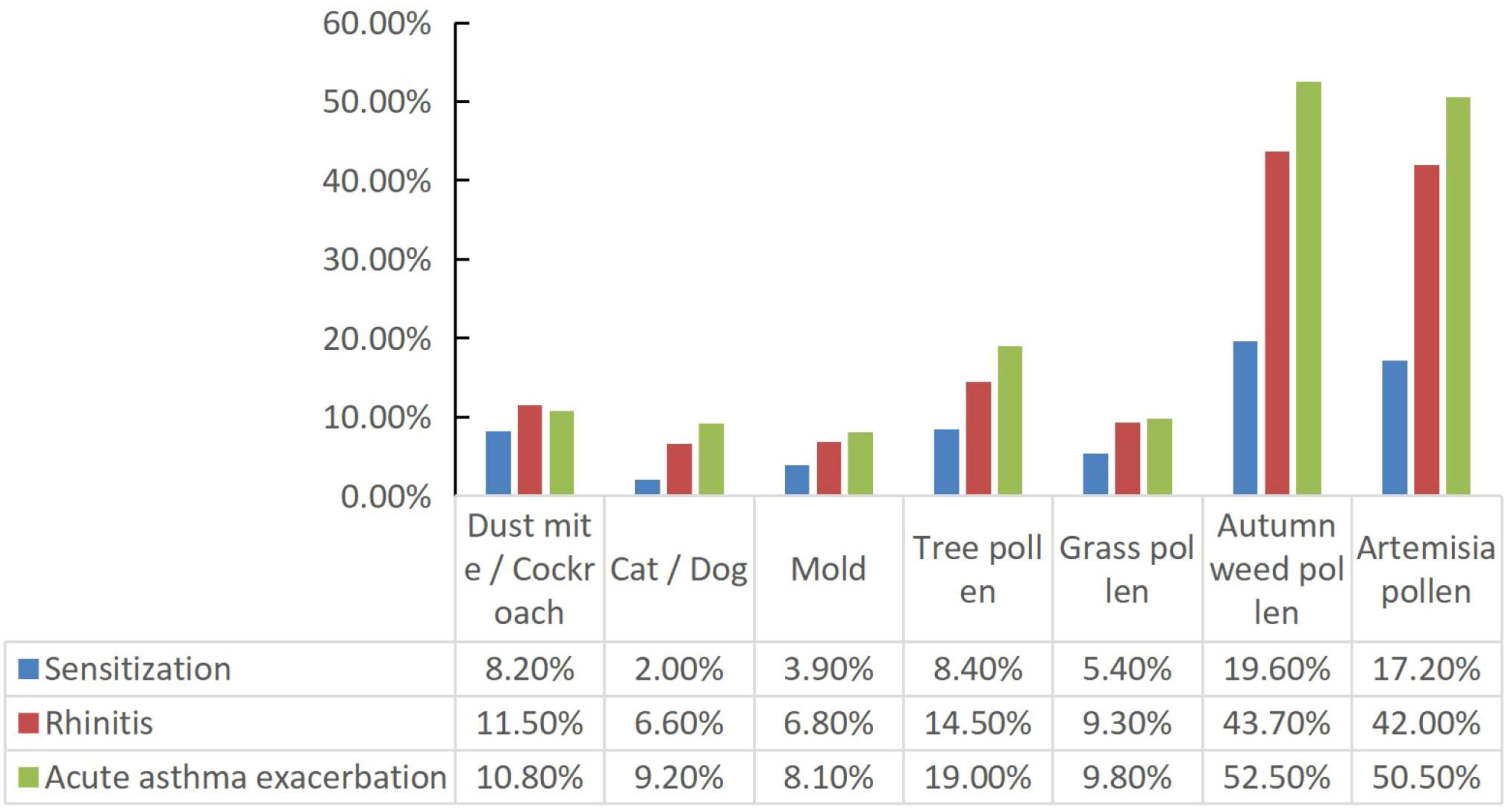
Sensitization rates to common inhaled allergens across study groups.

As shown in **Figure 1**, the sensitization rates varied across allergens. Autumn weed pollen showed the highest positivity rate (35.6%), with *Artemisia* pollen comprising 33.6%, followed by tree pollen (12.5%), dust mite/cockroach (10.4%), grass pollen (8.1%), mold (5.8%), and cat/dog (5.0%) showed lower rates.

**Figure 2:**
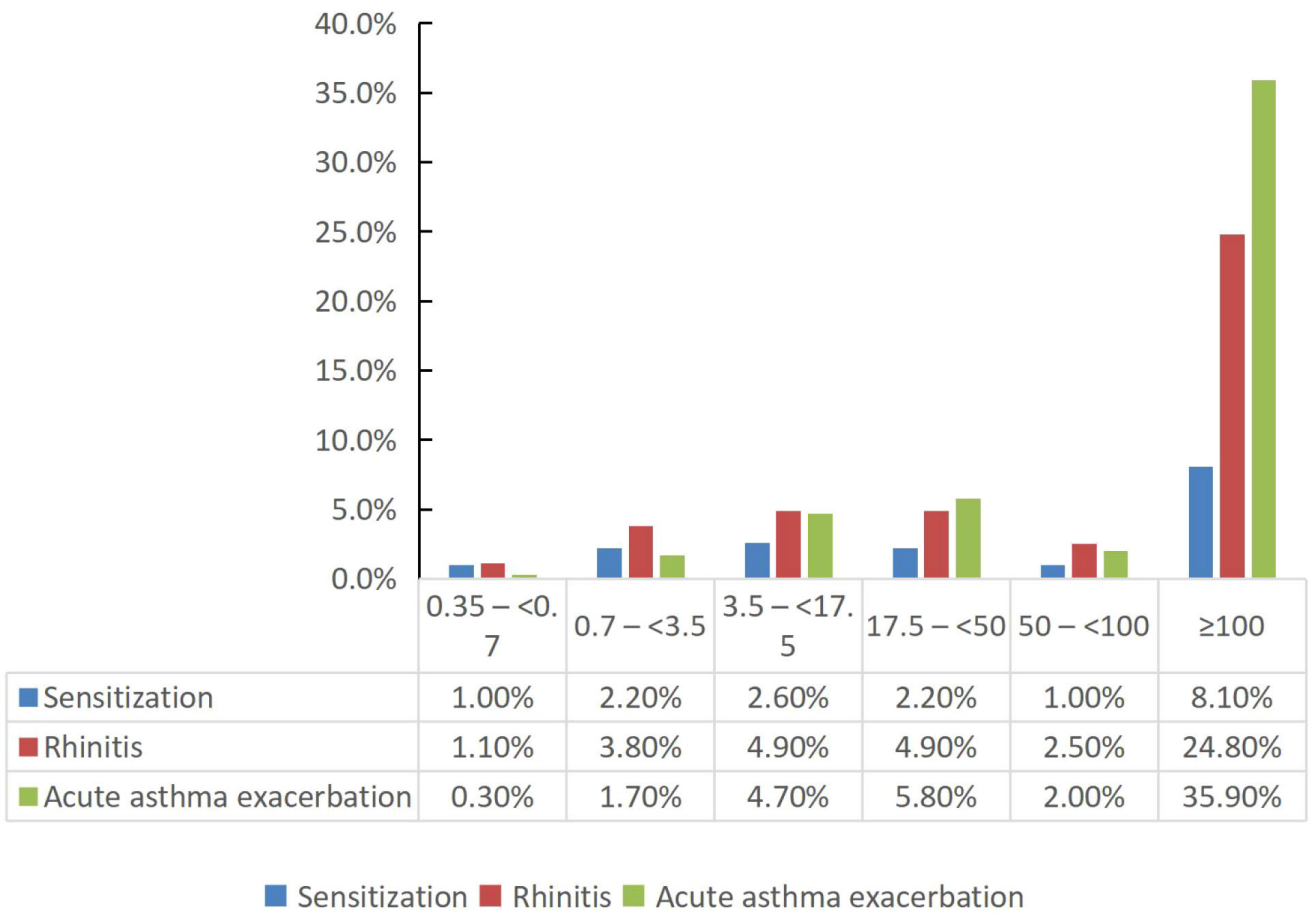
Distribution of *Artemisia*-specific IgE grades across study groups.

As shown in **Figure 2**, the distribution of *Artemisia*-specific IgE grades varied across the three groups. The proportion of participants with grade 6 IgE (≥100 kUA/L) was substantially higher in the acute asthma exacerbation group (35.9%) compared with the rhinitis (24.8%) and sensitization (8.1%) groups. Conversely, the proportion of IgE-negative individuals (grade 0) was lower in the acute asthma exacerbation group (49.5%) than in the rhinitis (58.0%) and asymptomatic groups (58.1%).

**Figure 3:**
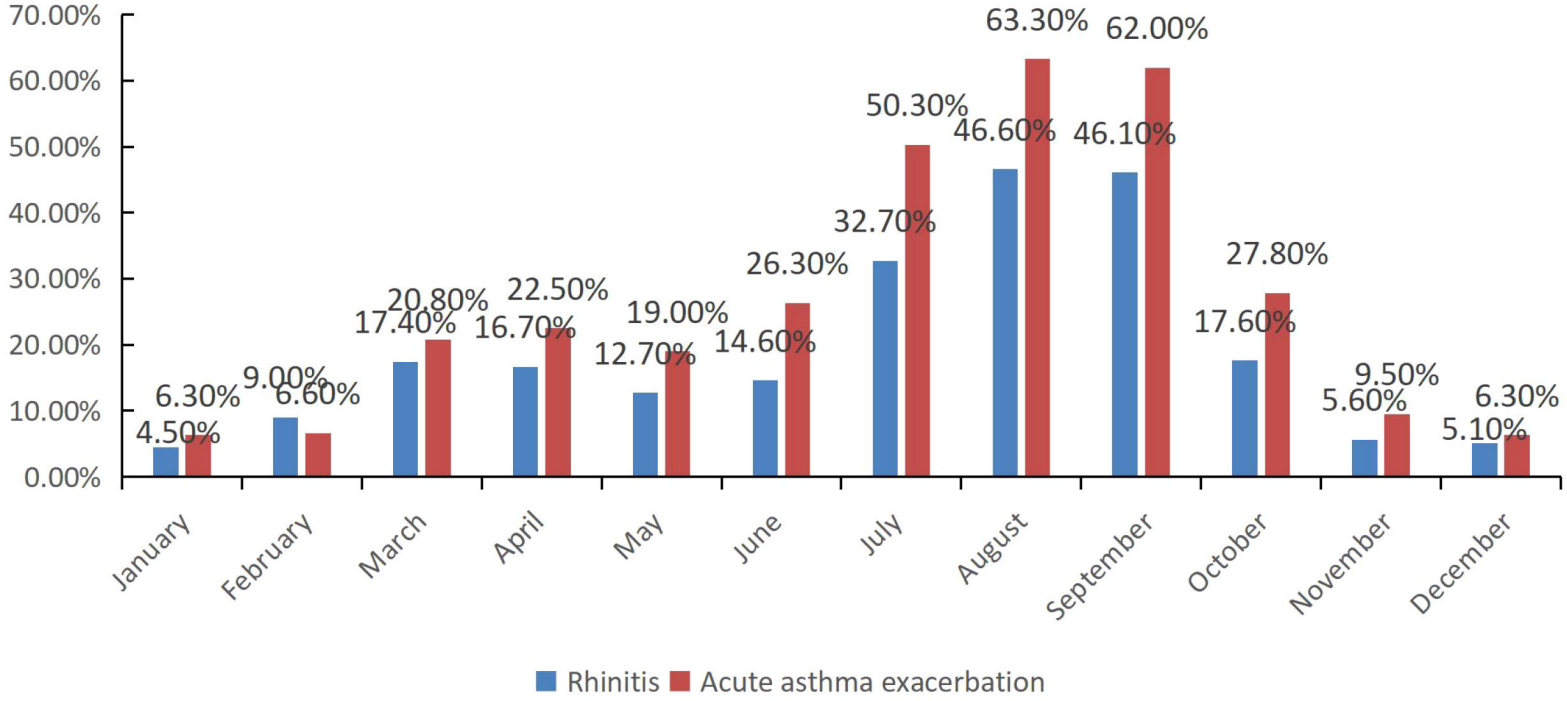
Seasonal distribution of allergic rhinitis and acute asthma exacerbations.

**Figure 3** shows that both AR and acute asthma exacerbations exhibited a typical bimodal seasonal distribution: the main peak occurred from July to October, and a secondary peak appeared from March to May. This seasonality closely aligns with the ecological characteristics of significantly elevated *Artemisia* pollen concentrations in Yulin during summer and autumn.

### 3.3 Inverse probability weighting-adjusted prevalence of *Artemisia*-induced sensitization, AR, and acute asthma exacerbations

To assess the representativeness of the blood-sampled participants, we compared their baseline characteristics with those of the non-sampled group (**Table 2**). The observed substantial differences confirm the presence of selection bias, which was subsequently addressed using inverse probability weighting (IPW) in all adjusted analyses. As shown in **Table 3**, among all 12,345 respondents, the IPW-adjusted prevalence of *Artemisia* sensitization was 13.0%, the prevalence of *Artemisia-induced* AR was 10.7%, and the prevalence of *Artemisia-induced* acute asthma exacerbations was 1.9%. Regarding the urban-rural distribution, the prevalence of *Artemisia-induced* AR was higher in urban residents (12.2% vs. 8.0%). Similarly, both the sensitization rate and the prevalence of acute asthma exacerbations were higher in urban areas (14.7% vs. 10.0% for sensitization, and 2.4% vs. 1.0% for asthma exacerbations). When stratifying by pollen exposure levels, the IPW-adjusted prevalence of acute asthma exacerbations in high-pollen areas (2.1%) was higher than in medium-pollen areas (1.1%) but lower than in low-pollen areas (2.4%). AR prevalence in high-pollen areas (12.3%) was higher than in both medium-pollen (8.4%) and low-pollen (11.1%) areas. The sensitization rate in high-pollen areas (13.8%) was higher than in medium-pollen areas (9.9%) but lower than in low-pollen areas (15.3%).

**Table 2:** Comparison of baseline characteristics between blood-sampled and non-sampled groups.

Characteristic	Subgroup	Non blood-sampled (N = 9,215)	Blood-sampled (N = 3,130)	P value
Pollen concentration	Low	2,524 (27.4%)	900 (28.8%)	0.063
	Medium	2,864 (31.1%)	905 (28.9%)	
	High	3,827 (41.5%)	1,325 (42.3%)	
Residence	Rural	3,639 (39.5%)	1,170 (37.4%)	0.037
	Urban	5,576 (60.5%)	1,960 (62.6%)	
Age (years)	Mean (SD)	39.11 (20.30)	36.03 (17.80)	<0.001
Sex	Male	4,378 (47.5%)	1,333 (42.6%)	<0.001
	Female	4,837 (52.5%)	1,797 (57.4%)	
Parental education	Low	5,404 (58.6%)	1,469 (46.9%)	<0.001
	Medium	2,229 (24.2%)	976 (31.2%)	
	High	1,582 (17.2%)	685 (21.9%)	
Family history of allergy	No	8,151 (88.5%)	2,277 (72.7%)	<0.001
	Yes	1,064 (11.5%)	853 (27.3%)	
Symptom status	Asymptomatic	7,901 (85.7%)	1,048 (33.5%)	<0.001
	Symptomatic	1,314 (14.3%)	2,082 (66.5%)	

**Table 3:** Inverse probability weighting-estimated prevalence of *Artemisia* pollen positivity.

Subgroup	*Artemisia*-induced sensitization (%)	*Artemisia*-induced AR (%)	*Artemisia*-induced acute asthma exacerbation (%)
Low pollen concentration	15.3	11.1	2.4
Medium pollen concentration	9.9	8.4	1.1
High pollen concentration	13.8	12.3	2.1
Rural	10.0	8.0	1.0
Urban	14.7	12.2	2.4
Total	13.0	10.7	1.9

Data represent IPW-adjusted prevalence in the overall population. AR, allergic rhinitis; IPW, inverse probability weighting.

### 3.4 Factors influencing *Artemisia* sensitization, AR, and acute asthma exacerbations

The results of multivariate logistic regression analysis performed on participants who completed serological testing (n=3,130) are shown in **Table 4**.

**Table 4:** Factors influencing *Artemisia* sensitization, allergy, and acute asthma exacerbations.

Variable	Sensitization vs. Healthy		Allergy vs. Sensitization		*Artemisia*-induced acute asthma exacerbation vs. allergic rhinitis	
	OR (95% CI)	P value	OR (95% CI)	P value	OR (95% CI)	P value
Pollen concentration		0.181		0.632		0.157
Medium vs. Low	0.697 (0.462–1.052)	0.086	1.126 (0.744–1.704)	0.576	0.607 (0.358–1.027)	0.063
High vs. Low	0.759 (0.526–1.095)	0.141	1.194 (0.829–1.719)	0.341	0.760 (0.497–1.160)	0.203
Residence (urban vs. rural)	1.080 (0.775–1.505)	0.649	1.284 (0.911–1.810)	0.154	1.367 (0.860–2.171)	0.186
Age	0.981 (0.972–0.990)	<0.001	0.997 (0.986–1.008)	0.574	1.006 (0.991–1.022)	0.412
Sex (female vs. male)	0.559 (0.411–0.761)	<0.001	1.216 (0.889–1.663)	0.221	1.366 (0.930–2.006)	0.112
Parental education		0.001		0.526		0.835
Medium vs. Low	1.393 (0.958–2.026)	0.083	1.117 (0.759–1.643)	0.576	1.048 (0.657–1.671)	0.845
High vs. Low	2.289 (1.500–3.494)	<0.001	0.881 (0.560–1.386)	0.583	1.178 (0.680–2.038)	0.559
Family history of allergy	0.913 (0.576–1.448)	0.7	4.568 (3.005–6.945)	<0.001	1.114 (0.763–1.627)	0.577
Log (*Artemisia*-sIgE)	—	—	1.176 (1.071–1.291)	0.001	1.244 (1.063–1.456)	0.006
Rhinitis severity	—	—	—	—		<0.001
Moderate vs. Mild	—	—	—	—	2.628 (1.325–5.210)	0.006
Severe vs. Mild	—	—	—	—	5.017 (2.558–9.839)	<0.001

CI, confidence interval; OR, odds ratio. — indicates the variable was not included in the model.

Compared with those with low parental education, those with high parental education had more than double the odds of sensitization (OR = 2.289, 95% CI: 1.500–3.494, P<0.001).

Each one-unit increase in Log *Artemisia*-sIgE was associated with a 17.6% increase in the odds of symptomatic allergy versus asymptomatic sensitization (OR = 1.176, 95% CI: 1.071–1.291, P = 0.001), and a 24.4% increase in the odds of acute asthma exacerbation versus rhinitis (OR = 1.244, 95% CI: 1.063–1.456, P = 0.006).

In the “*Artemisia*-induced acute asthma exacerbation vs. allergic rhinitis” comparison, pollen concentration showed no significant effect (overall P = 0.157). In exploratory piecewise linear regression analyses using a pollen concentration cut point of 150 grains/thousand mm², no statistically significant associations were observed between pollen concentration and any of the three outcomes in either the low-to-moderate (≤150) or high (>150) exposure strata (**Supplementary Table 2**). A marginal trend toward a protective effect of increasing pollen concentration on sensitization was noted only in the lower-exposure stratum (OR = 0.994, 95% CI: 0.989–1.000, P = 0.058), which did not reach statistical significance (**Supplementary Table 2**). Instead, rhinitis severity was the strongest predictor, with moderate rhinitis (OR=2.628, 95% CI: 1.325–5.210, P=0.006) and severe rhinitis (OR=5.017, 95% CI: 2.558–9.839, P<0.001) each showing significantly higher odds compared with mild rhinitis,, followed by *Artemisia*-specific IgE (Log *Artemisia*-sIgE; OR = 1.244, 95% CI: 1.030–1.337, P = 0.016).

## 4 Discussion

### 4.1 Disease burden of *Artemisia* pollen allergy

This large population-based cross-sectional study evaluated *Artemisia*-related sensitization and allergic disease across clinical stages in a high-exposure region of northern China. After adjustment for selection bias using inverse probability weighting, the prevalence of sensitization, allergic rhinitis, and acute asthma exacerbations was 13.0%, 10.7%, and 1.9%, respectively.

Compared with previous domestic studies, the prevalence of *Artemisia*-induced AR in Yulin (10.7%) is at a relatively high level, similar to rates reported in epidemiological surveys conducted in the grassland areas of Inner Mongolia (5), which aligns with the region's semi-arid climate and *Artemisia*-dominated vegetation (6). Notably, the proportion of *Artemisia* pollen positivity increased progressively from the sensitized population (17.2%) to the rhinitis population (42.0%) and further to the acute asthma exacerbation population (50.5%) (**Figure 1**), a trend that strongly supports the role of *Artemisia* pollen as a core driver of the allergic process in this region. The 2.1% acute asthma exacerbation rate in high-exposure areas is comparable to reports from some high-incidence pollinosis regions in Europe (13).

### 4.2 Interpretation of pollen exposure findings

The absence of a consistent association between regional pollen concentration and allergic outcomes should be interpreted in the context of ecological exposure assessment. In this study, pollen exposure was estimated at the regional level and may not accurately reflect individual-level exposure variability, including indoor environments, occupational exposure, and mobility patterns. This exposure misclassification may have attenuated potential associations toward the null.

The lack of association does not imply that pollen exposure is irrelevant, but rather that it may not be the primary driver of disease progression in this high-exposure setting. In a region such as Yulin, where *Artemisia* pollen exposure is universally high, exposure levels may have exceeded a saturation threshold, such that individual differences in exposure no longer determine disease risk. Once the population is universally exposed to sufficient pollen levels, disease development is likely driven primarily by host factors—including genetic predisposition, immune response intensity, and upper airway inflammatory status—rather than by further variations in exposure intensity (14). This saturation phenomenon can be explained immunologically: in long-term high-exposure environments, the immune system may establish partial tolerance through mechanisms involving regulatory T cells (15), which may explain why higher exposure does not necessarily lead to proportionally higher sensitization or disease rates.

Supplementary piecewise linear regression analyses, stratified at 150 grains/thousand mm², did not reveal consistent associations between pollen concentration and allergic outcomes across any stratum (**Supplementary Table 2**). A marginally significant inverse trend was observed for sensitization in the lower-exposure stratum (OR = 0.994, 95% CI: 0.989–1.000, P = 0.058), which did not reach statistical significance.

Overall, the present data do not support a stable dose–response relationship between regional pollen concentration and allergic disease outcomes in this population. However, these results should not be interpreted as evidence that pollen exposure is not biologically relevant, as it remains a necessary environmental trigger for sensitization in susceptible individuals.

### 4.3 Host susceptibility and disease heterogeneity

Host-related factors demonstrated more consistent statistical associations across analytical models compared with regional pollen exposure. Family history of allergy was strongly associated with the presence of symptomatic AR versus asymptomatic sensitization (OR = 4.57, 95% CI: 3.01–6.95), highlighting the central role of genetic susceptibility. However, this association was not observed in the comparison between sensitization and healthy controls, nor between acute asthma exacerbation and rhinitis, suggesting that genetic predisposition may play a more prominent role in the transition from sensitization to symptomatic rhinitis than in the initial sensitization process or in the further progression to asthma exacerbation. Possible mechanisms include specific gene polymorphisms (e.g., IL-4, IL-13, FCER1A) that may influence the intensity of immune responses to allergens, production of inflammatory mediators, and target organ sensitivity (16).

*Artemisia*-specific IgE levels showed a graded association across disease stages. Each one-unit increase in Log *Artemisia*-sIgE was associated with higher odds of symptomatic AR compared with asymptomatic sensitization (OR = 1.176, 95% CI: 1.071–1.291) and acute asthma exacerbation compared with AR (OR = 1.244, 95% CI: 1.063–1.456). **Figure 2** further shows that the proportion of high-grade (4–6) sIgE increased stepwise from asymptomatic sensitization to symptomatic rhinitis to acute asthma exacerbations. This dose–response relationship reinforces the value of specific IgE levels as biomarkers of disease activity and progression risk (11). In the acute asthma exacerbation vs. rhinitis model, rhinitis severity was the strongest predictor (OR = 2.628 for mild, OR = 5.017 for moderate/severe), followed by *Artemisia*-specific IgE, further supporting the concept of a unified airway in which upper and lower airway inflammation are closely interconnected.

In addition, parental education level emerged as a significant factor in the sensitization stage. Compared with those with low parental education, those with high parental education had more than double the risk of sensitization (OR = 2.289, 95% CI: 1.500–3.494), a finding consistent with the hygiene hypothesis (17). Families with higher parental education tend to have better sanitary conditions, smaller family sizes, less animal contact, and more indoor activity time, which may limit microbial exposure diversity during childhood and increase allergic sensitization risk. Notably, parental education showed no significant effects in the later progression stages, suggesting that socioeconomic factors may act mainly at the initiation of the allergic process rather than in its subsequent progression. These findings collectively support the role of host immunological responsiveness and early-life environmental exposures in shaping the heterogeneity of *Artemisia*-related allergic disease.

### 4.4 Limitations

Several limitations should be considered. First, the cross-sectional design precludes causal inference. Second, pollen exposure was estimated using regional monitoring data, which does not capture individual-level exposure variability. Third, although inverse probability weighting was used to reduce selection bias, residual confounding cannot be excluded. 

## 5 Conclusion

In this high-exposure population, the IPW-adjusted prevalence of *Artemisia* sensitization, allergic rhinitis, and acute asthma exacerbations was 13.0%, 10.7%, and 1.9%, respectively. Host-related factors showed more consistent associations across disease stages, whereas no consistent dose–response relationship was observed between regional pollen concentration and allergic outcomes. This study provides epidemiological evidence to guide future research into host susceptibility and immune mechanisms in pollen-related allergic disease.

## Data Availability

The datasets presented in this study are deposited in the epidemiological research module of the National Clinical Research Center for Dermatologic and Immunologic Diseases (https://www.allergists.cn/). Access to the data is restricted to protect participant privacy. Requests for data access may be directed to the corresponding authors and will be reviewed by the institutional committee.
