# Supplementary material for: Relative contributions of environmental pollen exposure and host susceptibility to Artemisia-related sensitisation and allergy in a high-exposure region of Northern China: a population-based cross-sectional study
Source: Front Immunol. 2026 Jul 23;17:1887300. doi: 10.3389/fimmu.2026.1887300 (PMC13442546; doi:10.3389/fimmu.2026.1887300)
Supplement: Supplementary file 1 [file DataSheet1.pdf]

Supplementary Table 1. Standardized mean differences (SMD) before and after inverse probability weighting (IPW) for key covariates

SMD values are presented as absolute values. |SMD| < 0.1 indicates good balance between groups.

Variable	Type	SMD Before (|SMD|)	SMD After (|SMD|)	Balance Achieved
Symptom status	Categorical	1.2583	0.0039	Yes
Family history of allergy	Categorical	0.4053	0.0314	Yes
Parental education	Categorical	0.2362	0.0159	Yes
Age	Continuous	0.1616	0.0129	Yes
Sex	Categorical	0.0990	0.0386	Yes
Pollen concentration	Categorical	0.0473	0.0188	Yes
Residence	Categorical	0.0434	0.0546	Yes

Supplementary Table 2. Factors influencing Artemisia sensitization, allergy, and acute asthma exacerbations:

Results of piecewise linear regression analysis (cut point = 150 grains/thousand mm^2)

Variable	Sensitization vs. Healthy OR (95% CI)	P value	Allergy vs. Sensitization OR (95% CI)	P value	Asthma vs. AR OR (95% CI)	P value
Pollen concentration (≤150 grains/thousand mm^2)	0.994 (0.989-1.000)	0.058 †	1.005 (1.000-1.011)	0.070 †	0.994 (0.987-1.001)	0.111
Pollen concentration (>150 grains/thousand mm^2)	1.004 (0.997-1.010)	0.274	0.995 (0.988-1.002)	0.171	1.005 (0.996-1.015)	0.244
Residence (Urban vs. Rural)	1.139 (0.816-1.590)	0.446	1.244 (0.880-1.758)	0.217	1.449 (0.912-2.303)	0.116
Sex (Female vs. Male)	0.563 (0.414-0.766)	0.000 ***	1.199 (0.876-1.641)	0.258	1.374 (0.935-2.020)	0.106
Age (per year)	0.981 (0.972-0.990)	0.000 ***	0.997 (0.986-1.008)	0.620	1.006 (0.991-1.021)	0.445
Family history of allergy	0.942 (0.592-1.498)	0.799	4.552 (2.993-6.921)	0.000 ***	1.118 (0.766-1.632)	0.564
Parental education (Secondary vs. Primary)	1.369 (0.937-2.000)	0.105	1.160 (0.785-1.712)	0.456	1.031 (0.645-1.647)	0.898
Parental education (University vs. Primary)	2.303 (1.509-3.515)	0.000 ***	0.892 (0.567-1.403)	0.621	1.165 (0.673-2.016)	0.585
Log(Artemisia-sIgE)	—	—	1.174 (1.069-1.289)	0.001 ***	1.243 (1.063-1.455)	0.007 **
Rhinitis severity (Moderate vs. Mild)	—	—	—	—	2.610 (1.317-5.172)	0.006 **
Rhinitis severity (Severe vs. Mild)	—	—	—	—	4.996 (2.548-9.795)	0.000 ***

*† P < 0.1; * P < 0.05; ** P < 0.01; *** P < 0.001*
